# How to measure staff continuity in intensive psychiatric home treatment: a routine data and single case analysis

**DOI:** 10.3389/fpsyt.2023.1166197

**Published:** 2023-05-09

**Authors:** Julian Schwarz, Jan Wolff, Martin Heinze, Sebastian von Peter, Juri Luis Habicht

**Affiliations:** ^1^Department of Psychiatry and Psychotherapy, Immanuel Klinik Rüdersdorf, Brandenburg Medical School Theodor Fontane, Rüdersdorf, Germany; ^2^Center for Health Service Research Brandenburg, Brandenburg Medical School Theodor Fontane, Rüdersdorf, Germany; ^3^Faculty of Health Sciences Brandenburg, Brandenburg Medical School Theodor Fontane, Neuruppin, Germany; ^4^Peter L. Reichertz Institute for Medical Informatics of TU Braunschweig and Hannover Medical School, Hannover, Germany; ^5^Evangelical Foundation Neuerkerode, Braunschweig, Germany; ^6^Department of Neurology with Experimental Neurology, Charité – Universitätsmedizin Berlin, Berlin, Germany

**Keywords:** community mental health care, StäB, Stationsäquivalente Behandlung, continuity of care, assertive community treatment, crisis resolution teams

## Abstract

**Background:**

Intensive forms of outreach mental health care (IOC) such as crisis resolution or home treatment teams are increasingly implemented as alternatives to inpatient admission, providing recovery-oriented treatment at home at comparable costs and outcomes. However, one issue with IOC is the lack of continuity regarding staff members who provide home visits, complicating relationship building and meaningful therapeutic exchange. The aim of this study is to validate existing primarily qualitative findings using performance data and to explore a possible correlation between the number of staff involved within IOC treatment and the service users’ length of stay (LOS).

**Methods:**

Routine data from an IOC team in a catchment area in Eastern Germany were analyzed. Basic parameters of service delivery were calculated and an in-depth descriptive analysis regarding staff continuity was performed. Further, an exploratory single case analysis was conducted, presenting the exact sequence of all treatment contacts for one case with low and one with high staff continuity.

**Results:**

We analyzed 10.598 face-to-face treatment contacts based on 178 IOC users. The mean LOS was 30.99 days. About 75% of all home visits were conducted by two or more staff members simultaneously. Service users saw an average of 10.24 different staff per treatment episode. On 11% of the care days, only unknown staff, and on 34% of the care days at least one unknown staff member conducted the home visit. 83% of the contacts were performed by the same three staff members and 51% were made by one and the same staff member. A significant positive correlation (*p* = 0.0007) was found between the number of different practitioners seen by a service user in the first seven days of care and the LOS.

**Conclusion:**

Our results suggest that a high number of different staff in the early period of IOC episodes correlates with an extended LOS. Future research must clarify the exact mechanisms of this correlation. Furthermore, it should be investigated how the multiple professions within IOC teams influence the LOS and the quality of treatment and what quality indicators may be suitable to ensure treatment processes.

## 1. Introduction

Internationally, intensive forms of outreach mental health care (IOC) have been implemented in various countries in recent decades and include, but are not limited to crisis resolution teams (CRT), flexible assertive community care (FACT) and home treatment (HT) (1–3). They are increasingly implemented as alternatives to inpatient admission, providing recovery-oriented treatment at home at comparable costs and outcomes (1–3), and are mostly provided by multi-professional teams in the users’ living environment. Research on IOC shows mainly positive effects, such as a decrease in symptom severity, inpatient treatment days, and discontinuation of treatment (4–6), while costs remain the same or decrease compared to inpatient admission (4, 7). One of the key issues of intensive IOC, on the other hand, is the lack of staff continuity during single care episodes (8–15). While in the inpatient setting, usually several members of a multi-professional team are simultaneously present on the ward for several days in a row, contacts in IOC are often provided by practitioners who change on a daily basis. Further, not all professions are represented every day, which is often the case in the hospital (10, 11, 15, 16).

A continuous relationship between user and practitioner is described as a key principle of psychiatric care (14, 17). In a widespread definition, this element of continuity of care (CoC) is named *relational continuity* and described as “*the therapeutic relationship between a patient and one or more clinicians* […] *and provides coherence through clinicians’ growing comprehensive knowledge of the patient*” (18). CoC defined by service users similarly emphasizes the significance of personal relationships with the same carer or contact person (19, 20). This work will focus on staff continuity, which we define as the same staff carrying out contacts with service users in a coordinated and consistent manner. Haggerty et al. name this type of continuity management continuity, which is also understood as a prerequisite for the perception of relational continuity (18). In addition to staff continuity, the term “team continuity” is frequently used, indicating that service users are cared for by members of the same treatment team (21). The evaluation of the impact of staff continuity on users’ outcomes shows contradicting results (22–25). It seems to be difficult to operationalize CoC (26) and there are only few and not widely used outcome measures (27).

On the one hand, studies on crisis resolution teams (CRT), a concrete model of IOC, conclude that staff continuity is crucial to service delivery (9, 10, 14, 28, 29). On the other hand, service users report to have seen too many staff involved in each episode of care, with contacts often being fleeting, superficial and requiring them to repeat their story several times (3, 8, 10, 29–32). A recent survey of patient satisfaction among users of psychiatric Home-Treatment (HT) identified poor staff continuity as one of the key sources of dissatisfaction (33). Although best practice guidelines and a fidelity scale for CRTs demand a named key worker, about which service users should be informed (14, 34), a recent implementation study carried out in 75 CRTs in the UK shows an average of poor fidelity to the model for staff CoC, indicating a need for improvement (35).

Few articles have analyzed the objective realization of staff continuity in IOC teams (11–13). One descriptive study found that service users of a CRT saw around five different staff members in one treatment episode lasting an average of 15 days (11). Another study shows an increase of staff continuity after having implemented a key worker system within an HT team (13). Williams et al. found moderate compliance with CoC criteria according to the CRT Fidelity Scale with only 55% (55/100) of service users being visited by their key worker at least once and 67% being informed of their key workers name (12).

Yet it is unclear which parameters are best suited to measure staff continuity in the IOC setting, and whether poor staff continuity affects the course of treatment, whether it prolongs the length of stay (LOS) or even leads to treatment discontinuation. We therefore aimed to quantitatively explore the staff continuity in one IOC team and to conduct an in-depth analysis on staff continuity regarding individual cases. The following research questions were examined:

1. How can staff continuity in an intensive outreach team be best assessed using routine data and illustrated in the individual case?
2. How does continuity of care within the first seven days of the home treatment impact the length of stay of patients in a psychiatric home treatment setting?

## 2. Methods

### 2.1. Design

Based on the hypothesis that high staff continuity is an indicator of high quality outreach mental health care, it should also be easily determined, preferably routinely collected. Based on this, the present study uses hospital routine data to explore appropriate parameters for measuring staff continuity in an IOC team and possible correlations to treatment outcomes. In order to understand the (non-) occurrence of continuity in greater detail, two extreme cases—one with high and another with low staff continuity were subjected to a single case analysis.

The data used in the present study were collected and evaluated for quality assurance purposes. In order to be able to use the results for research matters, they were anonymized in accordance with the Brandenburg Hospital Development Act (BbgKHEG). The existence of the vote of a medical ethics committee is therefore not required.

### 2.2. Context and setting

Since 2018, a federal law in Germany has allowed all psychiatric hospitals with a regional care obligation to implement so-called inpatient equivalent home treatment (IEHT; ger. “Stationsäquivalente Behandlung,” shortform: “StäB”) a particular form of IOC (36–39). Since then, about 60 of the totals of about 400 psychiatric clinics and psychiatric departments at general hospitals have implemented this new form of treatment with an increasing trend.

This study was conducted based on routine data from one psychiatric hospital in the federal state Brandenburg, Germany: The clinic of psychiatry and psychotherapy of the Immanuel hospital Rüdersdorf serves approximately 255,000 inhabitants and borders Berlin to the east. It is therefore responsible for a mixed urban and rural catchment area. IEHT was introduced at the study clinic in April 2018. It is provided by a multi-professional team consisting of ten employees or 5.8 full-time equivalents (FTE; as of January 2021). The IEHT team operates in two sub-teams, each responsible for a certain part of the catchment area. The total caseload per staff (not FTE) is around eight to ten service users; treatment contacts are usually carried out in pairs. Treatment contacts were exclusively face-to-face contacts (no video, phone, or text messaging).

### 2.3. Data collection

Routine data for the period from July 1^st^, 2018 to June 30^th^, 2021 were provided at the study center from the hospital information system (HIS). Besides socio-demographic case data, the data set included a detailed performance documentation of all therapeutic services provided, including date, time, duration, and professional group of the services delivered. These data must be collected annually by all hospitals in Germany and transmitted to the Institute for the Hospital Remuneration System. The information on the practitioners present at the service users’ home on each care day is not included in this data set but could be supplemented with the help of the hospital’s own documentation from the HIS. All data used were already completely anonymized at the time of provision.

### 2.4. Data analysis

Data were processed using a MySQL database (Oracle Corporation, Austin/Texas, United States) and basic parameters of service delivery were calculated. To determine staff continuity, we assessed several variables such as the number of practitioners involved within each treatment episode or the portion of care days delivered by unknown practitioners, i.e., care days on which service users have seen practitioners for the first time. Descriptive statistics were performed using GraphPad Prism version 9.0.0 for Windows (GraphPad Software, San Diego, California United States). As unpublished findings from a qualitative study on staff continuity within IOC teams indicated a relationship between high staff turnover in the first days after admission and the course of treatment (36, 37), a linear regression analysis was carried out to investigate the potential relationship between the number of practitioners seen in the first seven days of treatment and the total length of stay. We assumed a possible cause-effect relationship that we wanted to test and quantify, so regression analysis is the method of choice. We used the seven-day limit in calculating the staff turnover because extending the analysis to an extent where the total treatment period of all service users was included would naturally produce a positive correlation as the probability of coming into contact with different carers statistically increases within a longer period of time. Rather, our analysis aims to see if the number of different staff at baseline/at the beginning of the IOC affects the overall length of treatment.

Two cases were selected for the single case analysis, one of which has seen a very large and the other a small number of different practitioners within their IEHT care episode. In order to allow basic comparability between cases and to keep confounding factors low, a systematic case selection was performed: (1) all cases with an equal LOS were selected, (2) from these, pairs were again identified which were (a) by as different a number of involved staff as possible and (b) differed as little as possible with regard to sociodemographic parameters (gender, age in years as well as primary, secondary psychiatric and somatic diagnoses). For reasons of practicability, case selection was performed manually with the help of spreadsheet software, without using a specific method such as propensity score matching. For each of the two cases, it was then plotted for each care day when only unknown, partly unknown or known practitioners carried out the treatment contact. Furthermore, it was illustrated in a similar way which specific practitioner of which professional group was present on each individual treatment day.

## 3. Results

### 3.1. Basic parameters of service delivery

10.598 personal treatment contacts with IEHT staff members, 5.511 home visits, and 178 treatment cases were delivered in the observational period by the IEHT team of the study hospital. The socio-demographic information of the sample can be found in Table 1. Somatic diagnoses included all diagnoses excluding the ICD-10 F codes (i.e., mental, behavioral, and neurodevelopmental disorders). The mean length of stay (LOS) was 30.99 days (*SD* = 21.72). 87.08% of all service users were approached at home once a day; 12.92% had more than one contact with the IEHT team on individual days. 56.37% of all visits were delivered by two employees at the same time, 25.06% were carried out by just one person, and in 18.56%, three or four team members were present in the service users’ home. On average, 4.83 (*SD* = 1.12) different occupational groups were involved in a treatment episode, with nursing care (49.59%) providing the majority of all contacts, followed by psychiatrists (18.13%), social workers (14.94%) and peer support workers (10.18%).

**Table 1:** Sociodemographic characteristics of the sample (*n* = 178).

Parameter	Value
Gender *n*, (%)	
Female	112 (63)
Male	66 (37)
Age (years) mean, (*SD* )	53.94 (18.60)
Primary diagnosis *n*, (%)	
F0 (Organic, including symptomatic, mental disorders)	8 (4.55)
F1 Mental and behavioral disorders due to psychoactive substance use	3 (1.70)
F2 Schizophrenia, schizotypal and delusional disorders	53 (30.11)
F3 Mood (affective) disorders	85 (48.30)
F4 Neurotic, stress-related and somatoform disorders	21 (11.93)
F5 Behavioral syndromes associated with physiological disturbances and physical factors	0 (0.00)
F6 Disorders of adult personality and behavior	6 (3.41)
Secondary psych. diagnoses	
*n*, (%)	108 (60.67)
Mean, (*SD* )	1.17 (1.42)
Somatic diagnoses	
*n*, (%)	127 (71.35)
Mean, (*SD*)	2.60 (1.82)

*SD*, standard deviation; somatic diagnoses include all diagnoses excluding the ICD-10 F codes (i.e. mental, behavioral, and neurodevelopmental disorders).

### 3.2. Quantitative findings

**Figure 1:**
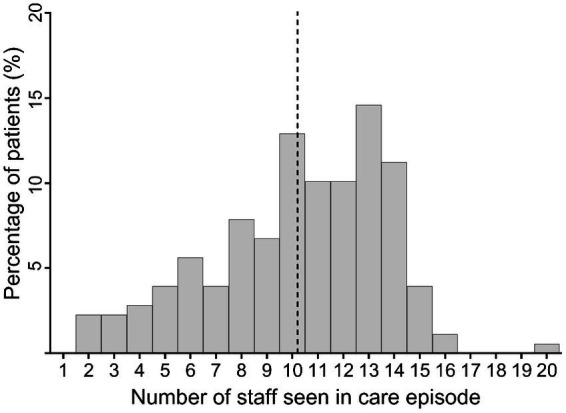
Histogram of number of different staff seen by a service user during care episode. The dashed vertical line marks the mean value of the number of practitioners seen in a treatment episode.

**Figure 2:**
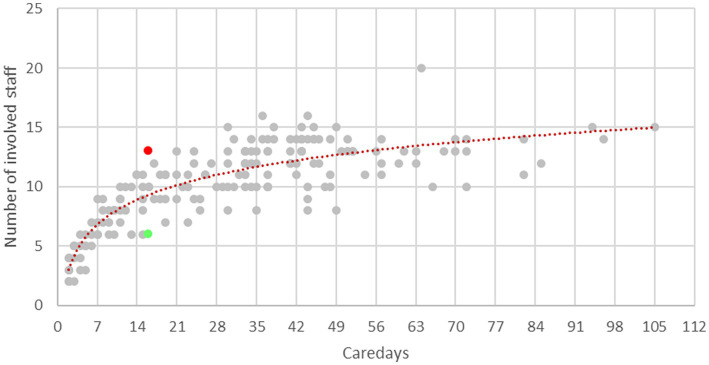
Number of staff involved in relation to the care days. Red dashed: logarithmic trend line showing the average number of practitioners involved in relation to the care days; marked in red: case with high staff turnover; Marked in green: case with low staff turnover.

Service users saw an average of 10.24 (*SD*  = 3.41) different practitioners within each treatment episode (see Figure 1). The number of practitioners in relation to the LOS is presented in Figure 2. The number of newly involved practitioners increases sharply in the first few days of treatment and then levels off with increasing LOS.

**Figure 3:**
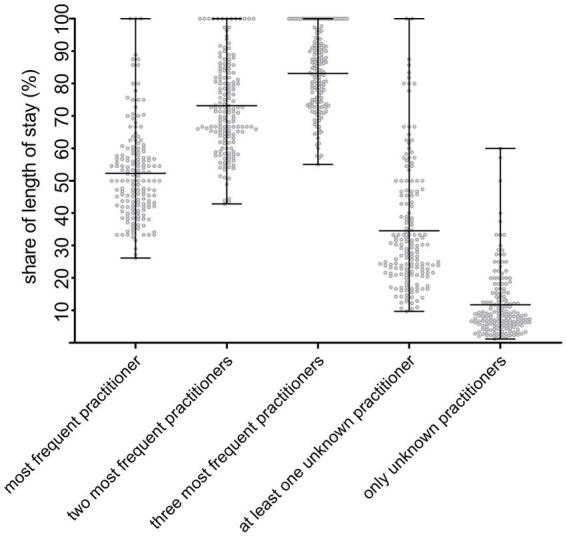
Performance of various parameters of staff continuity related to the length of stay. The proportion of home visits in relation to the entire treatment episode was calculated in which one and the same practitioner (“most frequent practitioner”) and the two or three most frequent practitioners (“two/three most frequent practitioners”) were involved. In addition, the proportion of home visits was determined in which at least one or only unknown practitioners were present (“at least one unknown practitioner” and “only unknown practitioners”). Each dot represents an individual case.

**Figure 4:**
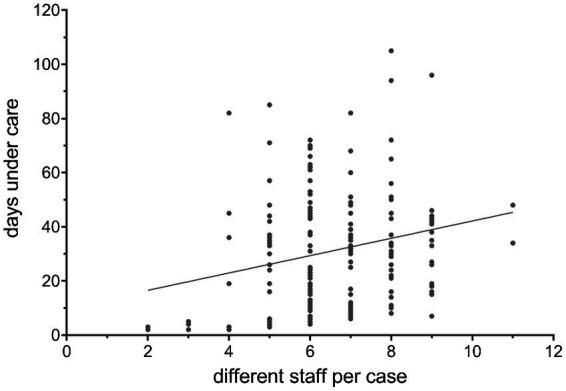
Correlation between number of different staff involved in the first seven care days and the care days. Each dot represents a single case. The ascending line shows the trend of the proportional relation between the number of different staff seen in the first seven care days and the lengths of stay using simple regression analysis.

On 2.52 days (*SD*  = 1.29) in each treatment episode only unfamiliar practitioners came to the users’ home; on 7.92 days (*SD*  = 3.10) at least one unknown practitioner was present during treatment. On average, 55.01% of all contacts within a treatment episode were delivered by the same team member, 74.63% by the same two or 85.27% by the same three practitioners. These and further parameters of staff continuity are visualized in Figure 3, each in relation to their share of the LOS. We analyzed the relationship between the number of different practitioners seen by a service user in the first seven days of care and the LOS using a regression analysis. As shown in Figure 4 and Appendix 1, a significantly positive slope (*P* = 0.00007) exists between those two variables.

### 3.3. Single case analysis

The two extreme cases with a particularly high (case A) and low staff turnover (case B), respectively, which were selected for the single case analysis, are shown as red and green dots in Figure 2. The socio-demographic characteristics of the cases can be found in Table 2.

**Table 2:** Sociodemographic characteristics of the cases selected for the single case analysis (*n* = 2).

Parameter	Case	A	B
Gender		Female	Female
Age (years)		51	57
Primary diagnosis (ICD-10)		F32.1	F32.1
Secondary psych. diagnoses *n*		1	1
Somatic diagnoses *n*		2	1

**Figure 5:**
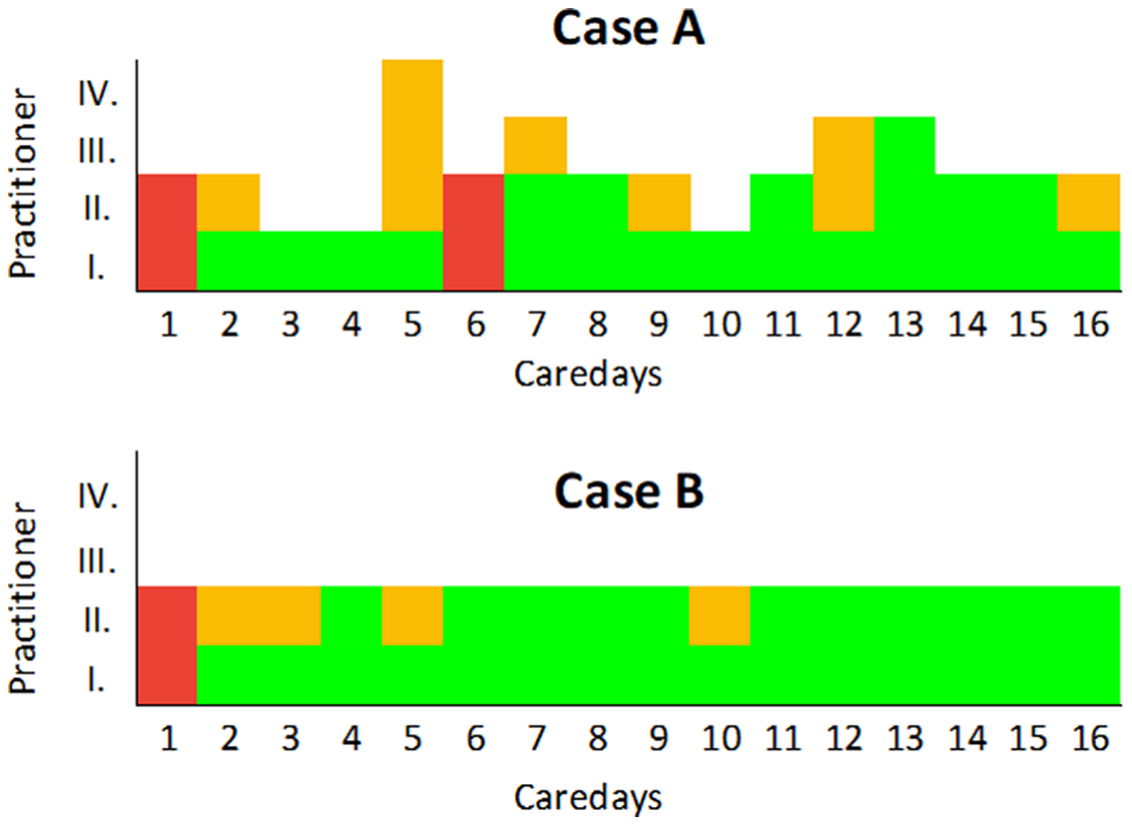
Care days with completely and partially unknown therapists in one case with a high (case A) and one with a low (case B) staff turnover. The care days are shown in the *X*-axis. In the two example cases shown here, only one home visit took place on each care day. The number of practitioners present at the same time during this visit is shown in the *Y*-axis. The fields marked in red indicate days on which only unknown practitioners were present. A field marked in yellow indicates a treatment contact with an unknown practitioner, and those marked in green indicate a contact with a known practitioner.

In case A, 50.00% (vs. 31.25% in case B) of all care days were delivered by at least partially unknown practitioners (see Figure 5). While care days with partially unknown therapists in case A are spread over the entire course up to the end of treatment, in case B, these contacts mainly take place in the first third of the treatment episode.

**Figure 6:**
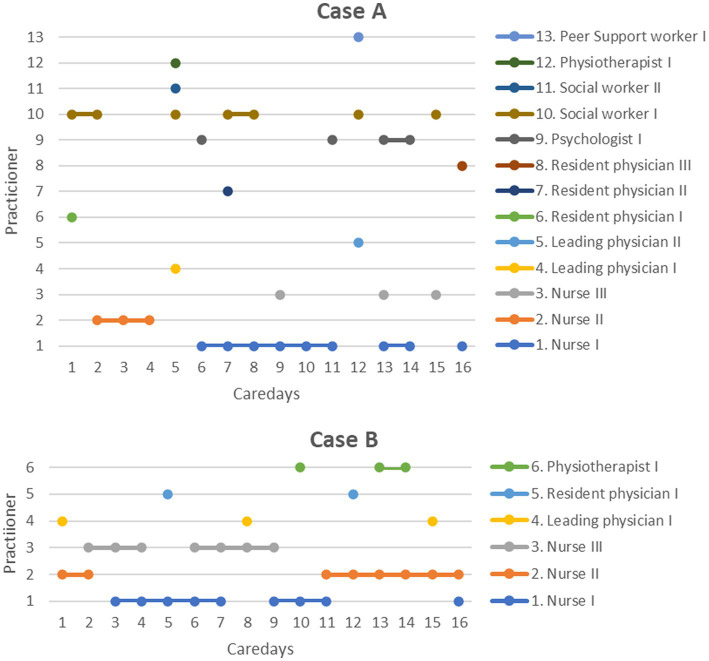
Presence of specific practitioners and professional groups in the course of treatment based on one case of a high number (case A) and one of a low number (case B) of involved staff members. The care days are shown in the *X*-axis. In the two example cases (cases A and B) shown here, only one home visit took place on each treatment day. All different practitioners involved in the treatment episode are shown in the *Y*-axis. Each dot represents a treatment contact with a certain individual and professional group.

A total of 13 different practitioners were present in case A; vs. six in case B (see Figure 6). After all, one and the same nurse was present on ning care days. Eight of the 13 practitioners only pay one visit in the entire treatment episode and do not pay another visit; this applies to all contacts with psychiatrists. Although all contacts are carried out by at least two people, 50% of the care days do not have any practitioners present who were already there the day before. In case B, all care days were carried out overlapping by three nurses, i.e., on each treatment day a nurse who was present the day before carries out the contact on the following day. In the other professional groups, each practitioner carries out at least two to three treatment contacts.

## 4. Discussion

In summary, the results show that CoC is a complex phenomenon: Depending on the aspects that are captured and how they are operationalized, i.e., which statistical method was applied to analyze them, a different picture of continuity emerges. Therefore, different conclusions can be drawn from a simple regression analysis (Figure 4) in comparison to an in-depth case analysis (Figure 5) and different aspects of practitioner (dis)continuity can be made visible. With an increase in the amount of information included per data set (= patient) within the different analyses, a more complex picture of staff continuity emerges. In order to depict the different aspects of continuity, different modes of representation are required, which depict the available data in a different degree of aggregation.

### 4.1. Principal findings

The fact that more than half of all contacts within a care episode take place in the presence of the same practitioner and that three recurrent practitioners are present on more than 80% of the care days suggests a high level of staff continuity in comparison with international studies (11–13). This is complemented by the finding that the number of days on which only unknown persons provide the treatment contact is rather low, averaging 2.5 days in relation to the mean LOS of 31 days. Moreover, these days are predominantly in the first third of the treatment episode, when staff are usually still unknown to the service user. To our knowledge, this parameter of staff familiarity has not been investigated in research yet, thus no comparable study results are available.

Statistically, at least one unknown practitioner is part of the home visit on every third day of the care episode which appears to be an obstacle to staff continuity in the present case. Existing studies show that the presence of a large number of unknown and alternating practitioners is experienced by service users as stressful, unsettling, and in some cases disruptive to the treatment situation, and stands in the way of the development of a recovery-oriented therapeutic relationship (3, 8, 10). This, in turn, has a negative effect on the service users’ satisfaction regarding the treatment (33).

The case analyses highlight that the sequence in which treatment contacts are delivered is central to the emergence of staff continuity. Future research needs to examine how long the interval between contacts with a particular staff member may be in order for an experience of continuity to emerge among service users. Our analyses also show the merits of having home visits conducted by two people instead of just one which led to an overlap between alternating practitioners (case B; see Figure 6), so that on each day at least one person was present who had already been present on the previous day. This approach could contribute significantly to informational and management continuity in addition to staff continuity. However, it is unknown how a multi-professional, more complex IOC involving more than two professions differs from outreach care that is only being carried out by one till two professions such as psychiatrists and nurses and how this affects the LOS. To our knowledge, there are no validated quality indicators to date for assessing IOC. Further (in the German context), no standard operating procedures exist yet to ensure that interprofessional IOC meets certain quality standards.

Our results suggest a highly significant association between the number of practitioners involved in the first seven days of treatment and the LOS. This contradicts the original hypothesis that a large staff turnover could overwhelm service users and thus lead to treatment discontinuation or a reduction in treatment duration.

An alternative explanation for our findings would be that service users who are more likely to see many different staff members in the first few days tend to need more time to develop a sustainable relationship, which, in turn, could have a prolonging effect on treatment duration. This is also consistent with existing qualitative findings that service users are more likely to experience a larger number of staff as stressful than beneficial (8–10, 15, 32, 33).

### 4.2. Measurement of staff continuity

In the present case, as is common in the literature (11–13), the number of staff involved per care episode was used as a central parameter for assessing staff continuity. This parameter has the advantage that it is easy to determine (based on the treatment documentation in the HIS) and allows a rough overview of staff continuity in a team. A prerequisite for this is that there is day-by-day documentation of all staff present at each contact with the service user. This was the case with the given data. It was also possible to calculate the proportion of contacts of certain staff members (“top treatment providers”) in an entire care episode without much additional effort.

In contrast, the number of care days with unknown service providers is more complex to calculate, since a complex integration of the data is necessary. In comparable studies, this parameter was not collected. It is important to mention that this parameter is only relevant if visits are performed by at least two persons together. If IOC contacts are performed by only one person, the number of contacts with unknown persons is usually equal to the total number of practitioners involved. Furthermore, this parameter assumes that all treatment providers are not yet known to the patient (e.g., from previous care episodes) at the beginning of treatment. In reality, this is often not the case.

In summary, it should be noted that all of the aforementioned parameters can only be evaluated together with the LOS when the number of treatment contacts increases and the number of staff involved also increases until all staff are known.

From our point of view, the individual case analysis seems to be suitable for quality management purposes or for optimizing care planning, as it provides insights into the concrete causes of discontinuity of care. würde ich die Reihenfolge zum besseren Verständnis ändern: The selection of extreme cases with the lowest vs. highest number of involved practitioners also allowed to improve the CoC in the synopsis the derivation of practical measures.

### 4.3. Strategies to increase staff continuity in IOC teams

Based on our findings and the existing evidence, we discuss measures that could contribute to an increase in staff continuity in IOC teams:

1. A core team of three reference practitioners, who alternate—in the best case even overlapping—could contribute to CoC. This has been shown by our example as well as by the work of Clinch (13). In our case, there were ten employees with 4.8 full-time equivalent (FTE), which corresponds to an average of 0.5 FTE per employee. The literature recommends that reference therapists should work with a 1.0 FTE to ensure high attendance or availability in the IOC team (12). In this respect, there is room for improvement at the study center under review. Further, the first visit could be carried out by all three key practitioners, thus enhancing CoC in the first days which is essential for relationship-building between carer and service user.
2. Statistically, service users get to know the majority of the treatment providers in the IOC team studied here in the first two weeks of treatment. During that phase, many unfamiliar staff members come home, which then decreases as treatment progresses. Service users should be informed about this at the beginning or even better prior to the service in order to be able to adjust better. From the third week of treatment at the latest, the entire IOC team should be known to service users, and only in exceptional situations (e.g., substitutions due to illness) may an unfamiliar staff member conduct the home visit.
3. As discussed above, information continuity is another relevant dimension of treatment continuity, which is directly influenced by staff continuity. In our example (see Figure 6, case B), on only 50% of the days did a person come to the home visit who had been present the previous day. In IOC teams where contacts are conducted by only one staff member, this proportion is likely to be further reduced. To ensure that information continuity does not suffer as a result of low staff continuity, sufficient communication tools and an (hospital) information system should be used in an IOC team to ensure that sufficient information is available about the progress of treatment in the event of staff changes.
4. Telephone contacts and video contacts (blended care) are other options increasingly used in IOC to allow service users to stay in touch with the staff members they are familiar with, for example, when a physical home visit is not possible on selected days, or complementary to it (11).

### 4.4. Strengths and limitations

This is the first study to use hospital routine data to examine the issue of staff discontinuity in IOC teams in depth and with respect to a possible relationship between CoC and LOS. This dimension of treatment continuity can be examined particularly well in outreach teams, since every patient contact is usually documented and thus evaluable. This is much more difficult in inpatient psychiatric settings, since in addition to regular therapeutic appointments, there are usually countless contacts with service users that are not (or cannot be) documented, but presumably influence the perception of staff continuity to a considerable extent.

A possible limitation of the study is that the question was investigated at only one study center, which could make it difficult to generalize the results. This should be countered by the fact that the available data at other hospitals offering IOC in Germany are often not available because they cannot be exported from HIS. Furthermore, the primary goal was more of a methodological-exploratory nature, namely, to investigate how staff continuity in IOC teams can be studied using routine data and which parameters are suitable to map this. Therefore, a single-center analysis is a well-suited approach.

Unfortunately, no association between practitioner (dis)continuity and outcomes could be investigated in the context of the present work. However, this would be necessary to prove whether a low number of involved practitioners or a high staff continuity are indicators for treatment quality. Also not examined was the duration of each treatment contact, information that is very important for the occurrence of staff continuity (18). It is reasonable to assume that a longer contact, for example, every other day, may achieve greater depth than a daily drop-in visit. Future research should examine these aspects.

Another important factor that was not taken into account, at least within the quantitative analysis, is the case complexity: It is reasonable to suggest that there are many confounding factors that affect the LOS, e.g., the severity of the diagnosis or the number of additional diagnoses a service user has. In this research, no direct data regarding case severity was available. Future research should take this into account to derive “optimal” LOS depending on the disease while considering additional factors (such as other diagnoses, severity of the disease, and social factors).

## 5. Conclusion

The home is a very private and still rarely used place for acute psychiatric treatment, especially in Germany. For service users to have a good experience here, it is important that they are treated by a consistent core group of practitioners with whom they can build a meaningful relationship. The present study has shown which parameters can be used to map the continuity of staff in the treatment process based on hospital routine data. This should be regularly monitored at least on a random basis in IOC teams and upper limits should be defined for the number of staff involved in a treatment episode. These should be specifically included in existing best practice models and fidelity scales for IOC, which already call for the introduction of a key worker system to improve treatment continuity, but without further specification (14, 35).

## Data availability statement

The data analyzed in this study is subject to the following licenses/restrictions: The datasets generated and/or analyzed during the current study are not publicly available due to privacy restrictions but are available from the corresponding author on reasonable request. Requests to access these datasets should be directed to julian.schwarz@mhb-fontane.de.

## Ethics statement

Ethical review and approval was not required for the study on human participants in accordance with the local legislation and institutional requirements. Written informed consent for participation was not required for this study in accordance with the national legislation and the institutional requirements.

## Author contributions

JS designed the study and drafted the first version of the manuscript. JS and JH performed the calculations. JH and MH supervised JS. All authors critically reviewed and commented on the manuscript.

## Funding

Funded by the Brandenburg Medical School publication fund supported by the German Research Foundation and the Ministry of Science, Research and Cultural Affairs of the State of Brandenburg.

## Conflict of interest

The authors declare that the research was conducted in the absence of any commercial or financial relationships that could be construed as a potential conflict of interest.

## Publisher’s note

All claims expressed in this article are solely those of the authors and do not necessarily represent those of their affiliated organizations, or those of the publisher, the editors and the reviewers. Any product that may be evaluated in this article, or claim that may be made by its manufacturer, is not guaranteed or endorsed by the publisher.

## Abbreviations

CoC: continuity of care
CRT: crisis resolution teams
FTE: full-time equivalent
HIS: hospital information system
HT: home treatment
IEHT: inpatient-equivalent home treatment
IOC: intensive outreach care
LOS: length of stay
SD: standard deviation
