# Supplementary material for: How to measure staff continuity in intensive psychiatric home treatment: a routine data and single case analysis
Source: Front Psychiatry. 2023 May 9;14:1166197. doi: 10.3389/fpsyt.2023.1166197 (PMC10204706; doi:10.3389/fpsyt.2023.1166197)
Supplement: Supplementary file 1 [file Table_1.DOCX]

**Appendix 1** Tabular results of the simple regression analysis

| **Simple linear regression (tabular results)** | **different staff per case** |
| --- | --- |
|  | |
| **Best-fit values** |  |
| Slope | 0.01960 |
| Y-intercept | 5.909 |
| X-intercept | -301.5 |
| 1/slope | 51.02 |
|  |  |
| **Std. Error** |  |
| Slope | 0.005711 |
| Y-intercept | 0.2162 |
|  |  |
| **95% Confidence Intervals** |  |
| Slope | 0.008330 to 0.03087 |
| Y-intercept | 5.483 to 6.336 |
| X-intercept | -751.7 to -179.7 |
|  |  |
| **Goodness of Fit** |  |
| R squared | 0.06273 |
| Sy.x | 1.655 |
|  |  |
| **Is slope significantly non-zero?** |  |
| F | 11.78 |
| DFn, DFd | 1,176 |
| p value | 0.0007 |
|  |  |
| **Equation** | Y=0.01960*X+5.909 |
|  |  |
| **Data** |  |
| Number of X values | 178 |
| maximum number of  Y replicates | 1 |
| Total number of values | 178 |
| Number of missing values | 0 |
